# A Navigation Probability Map in Pedestrian Dynamic Environment Based on Influencer Recognition Model

**DOI:** 10.3390/s21010019

**Published:** 2020-12-22

**Authors:** Zhi Qiao, Lijun Zhao, Xinkai Jiang, Le Gu, Ruifeng Li

**Affiliations:** School of Mechatronics Engineering, Harbin Institute of Technology, Harbin 150001, China; 15B908042@hit.edu.cn (Z.Q.); 19s008071@stu.hit.edu.cn (X.J.); gule@hit.edu.cn (L.G.); lrf100@hit.edu.cn (R.L.)

**Keywords:** pedestrian pattern, trajectory analysis, social navigation

## Abstract

One of the challenging problems in robot navigation is efficient and safe planning in a highly dynamic environment, where the robot is required to understand pedestrian patterns in the environment, such as train station. The rapid movement of pedestrians makes the robot more difficult to solve the collision problem. In this paper, we propose a navigation probability map to solve the pedestrians’ rapid movement problem based on the influencer recognition model (IRM). The influencer recognition model (IRM) is a data-driven model to infer a distribution over possible causes of pedestrian’s turning. With this model, we can obtain a navigation probability map by analyzing the changes in the effective pedestrian trajectory. Finally, we combined navigation probability map and artificial potential field (APF) method to propose a robot navigation method and verified it on our data-set, which is an unobstructed, overlooked pedestrians’ data-set collected by us.

## 1. Introduction

Mobile robots have increasing requirements for environmental perception. Especially under complex conditions, robots are required to have a multi-dimensional understanding of the environment. Sensors are important for robot environment perception. Sensors can help robots achieve simultaneous localization and mapping (SLAM), and can detect and locate moving obstacles in the environment. Both Laser scanner [1] and RGB-D (Red-Green-Blue and Depth) camera [2] can perform SLAM and pedestrian detection well. Laser generally detects the position of the legs of a pedestrian. The advantage is that the detecting position is accurate, but the single information of the laser makes it easy to detect incorrectly under crowded pedestrian conditions. The advantage of RGB-D camera is that it has diverse information and can obtain a bounding box for pedestrians, but it is easily affected by occlusion problems and illumination conditions. Microwaves can also be used to perceive the environment [3]. Microwave sensors have the advantage of being able to operate independent of external illuminations and that they are not sensitive to the color of the scene they image.

In addition to obtaining accurate environmental and pedestrian information, the highly dynamic pedestrian environment requires robots to have a sufficient understanding of pedestrian groups. Robots need to execute fast algorithms to avoid dynamic pedestrians. The requirements for avoiding pedestrians can be achieved through two aspects: a better understanding of pedestrian groups, and a better understanding of environmental characteristics.

Social navigation can be characterized as the interaction of two objectives—reaching one’s goal and maintaining socially competent behavior. Competent pedestrian navigation requires robots to reason about human intentions [4], which can be characterized as the pair of an eventual destination and a plan for getting there. A human observer employs teleological reasoning [5] to infer the intentions of another human based on the choice of actions they perform. Conversely, when the intention is known, humans anticipate a predictable action that minimizes energy [6]—a straight-line path.

**Figure 1:**
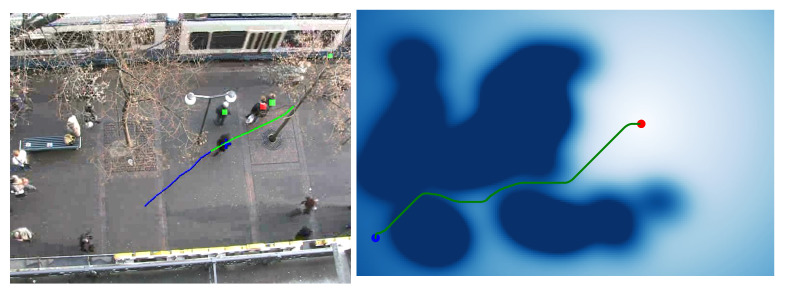
(**Left**): In a low-feature environment, a pedestrian’s turning is usually caused by other pedestrians (the person with red square). And others show their relative probability to be influencer with green square. (**Right**): Social navigation should avoid the areas that may affect pedestrians.

As the density of pedestrians in a space increases, the social competency objective increasingly conflicts with minimum-energy goal-attainment. To reconcile the conflict, humans plan more complex paths comprising sequences of subgoals, and people roughly follow a sequence of straight lines connecting subgoals. During social navigation in a pedestrian context, people employ teleological reasoning based on the observed sequence of motions in order to infer one another’s intentions (left in Figure 1). This inference allows people to anticipate the motions of others and select compatible motions in advance for collision avoidance.

In a specific environment, the trajectory of pedestrians will follow certain rules, which are related to the characteristics of the environment. This causes pedestrians to have preferences in the environment and get used to certain trajectories. In other words, certain areas of the environment are easier to gather pedestrians than others. These characteristics are important for understanding the environment and even for robot navigation. Social navigation should avoid the areas that may affect pedestrians (right in Figure 1). This will help the robot avoid pedestrians in a dynamic environment, because prior knowledge of the environment can be used to simplify navigation methods.

In this paper, we propose the influencer recognition model (IRM) to address the problem of identifying subgoals of a focus agent and predicting the cause of each subgoal based on observation of their path in context. And we propose a pedestrian-friendly robot navigation method that can understand environmental characteristics. Our first contribution is a model built from the interactive relationship between pedestrian trajectories, which is data-driven, probabilistic, and causative. Our second contribution is a robot navigation method that combines probability map and artificial potential field (APF). It learns environmental features through IRM models to obtain probability maps.

## 2. Related Work

Robots have a variety of technologies to sense the environment. Commonly used technologies include camera, laser and RGB-D camera, and so forth. Visual perception is currently the most widely used method for indoor environments. ORB-SLAM3 [2] is the first system able to perform visual, visual-inertial and multi-map SLAM with monocular, stereo and RGB-D cameras. YOLO [7] is a fast visual method to object detection, which frames object detection as a regression problem to spatially separated bounding boxes and associated class probabilities. For outdoor scenes, laser is more suitable as a mean of environmental perception. LAOM [1] is a real-time method for low-drift odometry and mapping using range measurements from a 3D laser scanner moving in 6-DOF, which achieves both low-drift in motion estimation and low-computational complexity. In addition to vision and laser, there are many other perception methods. Del Hougne [3] proposed the environment’s complexity enables solutions based on wave fingerprints (WFPs). Sacrificing diversity for SNR may be worthwhile and observe that simple artificial neural networks outperform traditional decoding methods in terms of the achieved sensing accuracy.

Collision avoidance is a widely explored problem in the field of robotics. For manufacturing robotic arms, Geismar et al. described a scheme that allows multiple robots to cooperate without colliding or suffering gridlock [8] (the robot is deadlocked and unable to choose action correctly). For service robots, Savkin et al. [9] proposed an algorithm for collision-free navigation of a non-holonomic robot in unknown complex dynamic environments with moving obstacles. Based on collision avoidance, the work in social navigation primarily addresses the related problems of the prediction of social agents and the control of the robot. The prediction methods can be divided broadly into global and local methods. The local method focuses on the analysis and simulation of single agent with local interactions and usually makes decisions based on little information. The global method in contrast considers the history and context of the interaction.

The best-known local method is the social force model proposed by Helbing and Molnar [6]. This method constructs attractive and repulsive forces to guide agents’ motions; pedestrians are repelled by one another and by static obstacles, whereas an attractive force towards the goal attempts to maintain progress. Other authors improved and expanded it variously. Tamura et al. [10] assumed a goal in the front of pedestrian and added a social force model to predict their trajectory. Linear trajectory avoidance (LTA) [11] is a pedestrian prediction method like the social force model in various aspects, but it differs in how to deal with agents. In LTA simulation, pedestrians have decisive directions and optimize paths by a collision-free principle, rather than just being reactive particles driven by force. Being derived from the social force model, all these methods ignore some valuable information given by past trajectories of the agents.

Next, we consider global methods, which use the entire trajectory history of a pedestrian to predict future trajectory. The pedestrian’s trajectory could be modeled as a Markov process. A single state (current and initial position) is sufficient to predict their motion in an environment with many static obstacles, where their trajectories are influenced greatly by the environment [12]. Pedestrians always choose a similar path from a common place to another one so that their trajectories are limited in such space. The Markov model breaks down in a low-clutter environment because pedestrian motions are influenced primarily by other pedestrians rather than the static environment, such as the scene in the BIWI walking pedestrian data-set [11]. This environment still has two obvious static obstacles in the camera’s view (the left and right snow boundaries), but the central region is nearly obstacle-free, so pedestrian-obstacle interactions are rare.

For conscious pedestrians, those who put most attention on walking, their historical trajectories and velocities are important information, and we can extrapolate their future trajectory from historical trajectories. Learning the motion patterns of persons and utilizing them is a common way to predict the action of people [13]. This approach collects persons’ motion data by laser-range sensors in an enclosed environment, then applies the EM (Expectation-Maximization) algorithm [13] to cluster pedestrians’ trajectories into motion patterns. Based on the result of the clustering patterns, they derived a hidden Markov model that is applied to estimate the current and future positions of persons. In an indoor environment, Ziebart et al. [12] also collected pedestrians’ trajectories over time and make path predictions with the principle of maximum entropy. The method obtains a cost-map of the current environment and estimates a distribution to predict possible future path from only the initial and current positions. These approaches are limited to known environments; pedestrian behaviors are also subject to factors that are unique to a given environment. The SBCM framework [14] predicts a pedestrian’s future trajectory by collecting trajectories in various conditions with a pedestrian ego-graph (PEG). This method takes advantage of general spatial effects (GSEs), which are conventions of pedestrian behavior that apply everywhere, and it discovers specific spatial effects (SSEs), which apply only in a specific context. The drawback of identifying GSEs and SSEs is that they neglect the interaction between pedestrians, which leads to missed information implied by subgoals. Zou et al. [15] introduced a scalable spatial-temporal graph generation adversarial network architecture. And they proposed the global node to integrate scene context information. But as the complexity of the environment grows, pedestrian behavior deviates increasingly from predictable trajectories by adding more subgoals. Most pedestrians will choose to walk a straight line in an open space because people always choose the shortest path to get to their destination. In a crowded environment, pedestrians will change their trajectories because of interaction with others. Most instances fall into two categories. Pedestrians change their trajectories to avoid someone in front of them or to return to their original trajectories after avoiding a pedestrian.

For control of a robot, Jur et al. [16] presented a formal approach to reciprocal n-body collision avoidance. This method is the first that can guarantee local collision-free motion for a large number of robots in a cluttered workspace. But the problem is that it only uses position and velocity information, without considering complex interactions. Chen et al. [17] presented a decentralized multi-agent collision avoidance algorithm based on a novel application of deep reinforcement learning. The input information of this work is same as reciprocal n-body collision avoidance, but its algorithm is more efficient. Then they extended this method and proposed a strategy using long-short term memory (LSTM) [18]. This algorithm outperforms their previous approach in simulation as the number of agents increases. Choi et al. [19] proposed a LSTM agent with Local-Map Critic (LSTM-LMC) and introduced the dynamics randomization technique to improve the robustness of the DRL agents in the real world. Although the above methods have achieved great results in terms of performance and efficiency, one of their common problems is that they only consider avoiding pedestrians without analyzing static obstacles. In other words, the characteristics of the environment are not considered.

## 3. Problem Statement

### 3.1. How to Find the Influencer?

**Figure 2:**
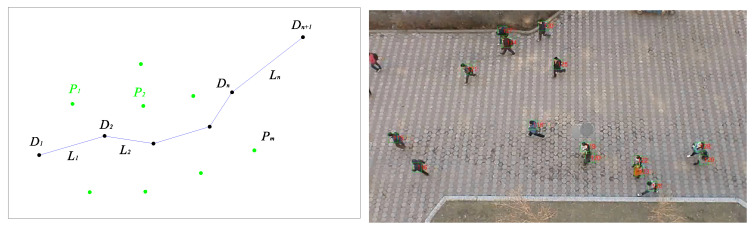
(**Left**): This figure shows this problem abstractly, black dots $D_{i}$ are deflection points of the focus agent and green dots $P_{i}$ are other pedestrians when D2 happens. (**Right**): Pedestrians usually follow certain common characteristics in an environment.

We are given a scene with a set of m pedestrians (left in Figure 2), $P_{j}=P_{1},P_{2},...,P_{m}$, one of whom is a focus agent F. Each pedestrian j is observed to follow a time-parametrized trajectory $f_{j}:R\Rightarrow R^{2}$ in the interval $0\leq t\leq t_{f}$. The focus agent’s trajectory $f_{F}$ deflects one or more times for unidentified reasons.

We articulate the following problems. First, we consider in Section 4 how to segment *F*’s trajectory properly to capture subgoals. Second, in Section 5 we seek a model to explain *F*’s deviations from a straight-line path at each subgoal by attributing them to a pedestrian influencer. Third, in Section 6 we evaluate the model’s performance. Finally, we summarize our research in Section 7 and ask how the model can be applied to the problems of predicting a focus agent’s destination and predicting influencing agents that are outside of the observer’s field of view.

### 3.2. Social Navigation with Environmental Characteristics

There is a typical pedestrian interaction environment (right in Figure 2). In this environment, many pedestrians choose different corridors to walk. However, after continuous observation, it will be found that most pedestrians will follow certain rules in this environment. Some corridors are chosen frequently by pedestrians. Furthermore, pedestrians will be more likely to influence each other in certain areas.

After finding these areas, how to make the robot avoid these areas as soon as possible while navigating is the second main problem we solve. We mainly describe the data-set, methods, and navigation results in Part 8.

### 3.3. A Camera-Robot System

**Figure 3:**
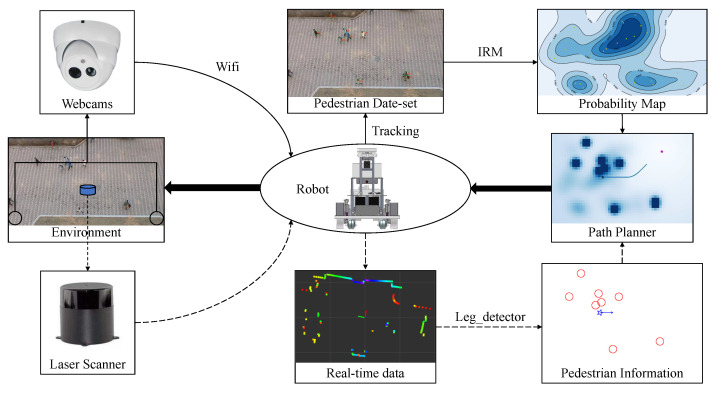
A system that can be used to research the problems we proposed. The webcams obtain a complete pedestrian data-set from a bird’s-eye view for trajectory analysis (Section 3.1). The robot uses laser scanner to obtain real-time pedestrian information for navigation in the crowd (Section 3.2).

Figure 3 is a system that can be used to research the problems we proposed. The solid arrows represent the process of learning pedestrian relationship and environmental characteristics, the dotted arrows represent the process of the robot itself perceiving the environment, and the bold arrows represent the process of robot control.

The bird’s-eye view camera, which is easy to obtain in various situations with webcams, is used to track the pedestrian trajectory in the environment, so as to research the pedestrian relationship (IRM) and environmental characteristics(probability map). After tracking and calibration, the image data obtained by the camera can be transformed into two-dimensional data. Some published data-set (such as BIWI Walking Pedestrians dataset [11]) can also be used for trajectory analysis. If there is a real-time requirement for environmental characteristics (for example, the current probability map will change as the density of pedestrians changes significantly), the webcams can transmit the data to the robot system for calculation through wifi.

The robot equipped with laser scanner can obtain its location through SLAM, and can obtain the current pedestrian position through “leg detector” in ROS (Robot Operating System) [20]. The leg detector package takes Laser Scans as input and uses a machine learning trained classifier to detect groups of laser readings as possible legs. The leg detector has another advantage about safety. The laser scanner works at a height of less than 1 meter, so it is not easy to scan the eyes of most people. Then the current information of pedestrians and the learned probability map can be combined to obtain a path planner, and the robot system can control the robot to navigate in the environment.

## 4. Trajectory Segmentation and Labeling

We focus here on pedestrian navigation in environments with low levels of obstacle clutter. Therefore, any deflection of a pedestrian’s trajectory is usually due to collision avoidance with other pedestrians. Thus, the key question is which pedestrian is influencing the focus agent’s trajectory when the deflection occurs. A consistent and rigorous method is needed for determining where the deflection occurs in an arbitrary focus agent’s trajectory. Trajectory segmentation is also a way for the robot to understand the trajectory of pedestrians. After the pedestrian’s trajectory is divided, it is helpful to analyze the intention of the pedestrian.

There are many general methods for trajectory fitting. For example, Lee and Xu [21] present a derivation for a spline smoother that considers local velocity information. Such a method captures both the signal and measurement noise. We therefore fit a trajectory with straight lines, in order to fit the social model of piecewise-predictable trajectories. The sequence of lines in turn exposes the focus agent’s intention.

In this section, we describe our technique of curating the ETH overhead pedestrian data from Pellegrini, et al. [11], but the techniques apply to any similar data-set.

### 4.1. Trajectory Segmentation

In the segmented representation, the time-parameterized trajectory of F will be approximated by n piecewise linear segments $L_{i}:1\leq i\leq n$ bounded by n+1 points $D_{i}\in R^{2}$ at times $T_{i}$ for 1≤i≤n+1, each corresponding to a subgoal or deflection point (left in Figure 2). Meanwhile at each point $D_{i}$, the pedestrian i occupies position $f_{j}\left(t_{i}\right)$.

The continuum trajectory $f_{j}\left(t\right)$ of agent *j* is sampled in the ETH data-set at 2.5 Hz, giving positions in meters of all agents in the scene for each time step. The objective is to segment the trajectory of each focus agent such that piecewise linear segments approximate the trajectory well. If we had access to the continuum trajectories, the process would be simple:

1. find all the inflection points, i.e., $\frac{d^{2}}{dt^{2}}f_{j}\left(t\right)=0$
2. filter the inflection points to ensure they are sufficiently different, by removing one neighbor whenever adjacent inflection points differ by less than a threshold *Q* (we used $6^{\circ }$)
3. perform k-means clustering of points in $f_{j}\left(t\right)$ to the filtered inflection points
4. define a subgoal wherever two clusters meet, and connect consecutive subgoals by lines.

Here we briefly explain how we approximate the above algorithm on the data-set with sampled positions. To filter out small noise, we fit a straight line in a sliding-window fashion to every three consecutive points using linear regression, yielding a vector $A=[A_{1},A_{2},\ldots ,A(d-2)]$, comprising the sequence of angles between each line and the global x-axis. The idea is to find the local extrema in the angles. To do so, we construct skew-symmetric matrix U of the differences between pairs of angles $\left(A_{p},A_{q}\right)$. We mark any element in this matrix, whose four neighbors are either all greater or all less than it is a local extremum. Selecting these cells from the matrix is the discrete equivalent to solving for a derivative equaling zero. Each element U yields a pair of indices (row and column) each of which is a potential extremum. The set of all such candidate extrema indices is collected and sorted. From here, indices are eliminated by thresholding if they differ by less than $6^{\circ }$. Finally, all position samples in the trajectory are clustered using k-means, and the clusters form straight lines connecting the subgoals.

**Figure 4:**
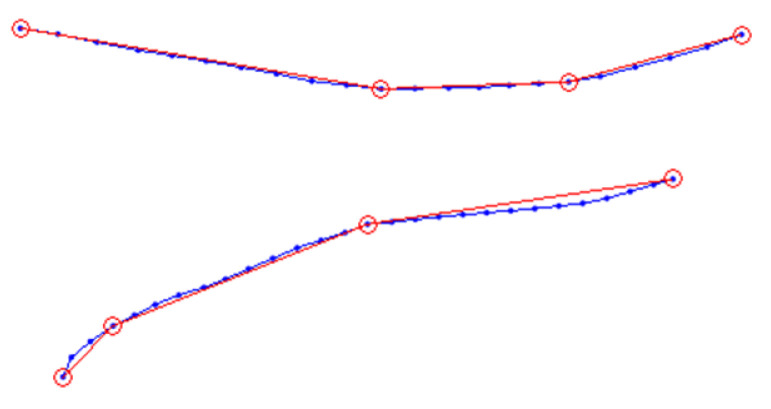
Two fitting-lines of pedestrians’ trajectories. Blue dots are tracking points and red circles represent subgoals calculated by fitting method. When the trajectory has a part with a small radian, this method will fit it inaccurately.

This approach solves the trajectory’s fitting-line problem successfully if the trajectory is approximately piecewise linear (upper Figure 4). As a global fitting method, it will not be influenced by subtle noise and we can adjust the fitting degree by controlling threshold *Q*. But if the trajectory changes gradually with small curvature, the approach will not work well because it is difficult to fit arc with line segments (lower Figure 4).

### 4.2. Labeling

We labeled by hand the influencers of focus agents by watching the video. Two rules guide our interpretation of cause and effect for agents whose intentions could be easily discerned. Outliers such as wandering agents, static agents, incompetent agents and groups were excluded.

The primary goal of labeling is to discern the reason that pedestrians change their trajectories. For every pedestrian, deflection may be caused by a static obstacle in the environment, by distraction, or a special pedestrian. If there is a special pedestrian influences the trajectory of focus agent, then record the number of the pedestrian and take him or her as an influencer.

The hardest part of the labeling process is how to ensure objectivity and uniformity through it. When the deflection is caused by another agent, it typically falls into one of two situations: (a) focus agents turn to avoid collision when they find another pedestrian in front of them (avoiding case); (b) focus agents deflect after passing another pedestrian who was blocking their way (returning case). These two cases are most often separate aspects of the same situation—people usually avoid first when they find pedestrians obstructing their path and then return to the original direction after passing the blocking pedestrian.

**Figure 5:**
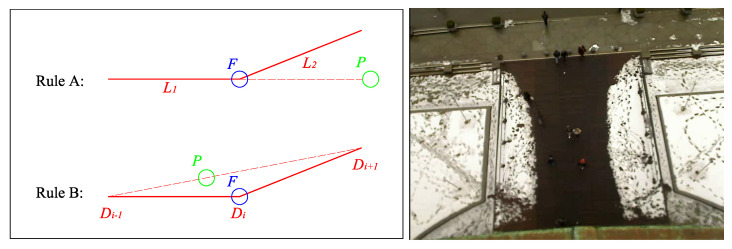
(**Left**): Two labeling scenarios: (top) in the avoiding case, F deflects to avoid pedestrian P (bottom) in the returning case, F finishes avoiding P and moves to the next goal. (**Right**): the training data-set [11].

From these two cases, we can conclude two rules for judging influencers (Figure 5): (a) if a pedestrian P is in front of the focus agent F and the original fitting-line $L_{1}$ would lead to a collision, then we regard the front pedestrian as the influencer; (b) if, when deflection $D_{i}$ happens, another pedestrian blocks the straight line from subgoal $D_{i-1}$ to subgoal $D_{i+1}$, then we regard the blocked pedestrian as the influencer.

According to the two rules above, we label the ETH data by watching the behaviors of every pedestrian in the video. There are 420 agents in the data-set. Of these, 178 trajectories are valuable samples. The others are missing data or have no interaction with other pedestrians.

### 4.3. Coordinate Transformation

To observe the relative position between the focus agent and influencer at the same time with subgoal $D_{2}$, we need to transform all the focus agents with one infection point to the same coordinate system. We transform the first fitting line L1 to negative Y-axis to make sure the focus agent come from −Y to +Y and move infection point $D_{2}$ to (0,0). Influencers’ positions will be transformed with the same matrix and the agents with left bend need to be mirrored to right bend.

**Figure 6:**
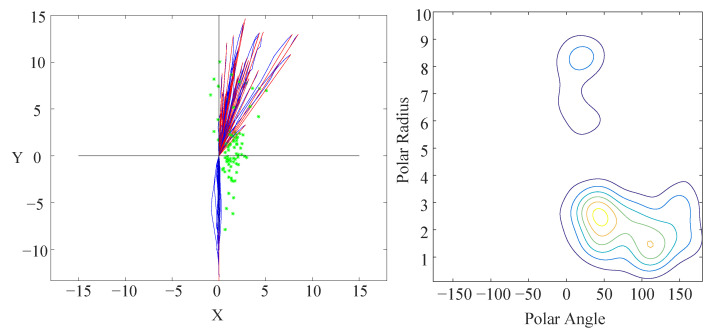
(**Left**): The positions of influencers when the corresponding focus agents turn. The green star is the influencer, the blue line is the focus agent’s trajectory, and the red line is the fitting-line. Most influencers gather around (2,0) and others cluster in front of the focus agent. (**Right**): The contour of the influencer probability distribution when the focus agent turns. The horizontal axis is the polar angle, and the vertical axis is the polar radius. Most influencers gather in the inner side of the turn, but a minority of other influencers gather in the front of the focus agent.

Now it is a map with respect to focus agents’ trajectories and the influencers’ positions when the subgoal $D_{2}$ happens (left of Figure 6). Many influencers gather around (2,0). People always turn after passing another pedestrian in the direction they are going to turn, just in case they will confuse others when they turn. Some other influencers appear in the front of the focus agent with an obvious explanation. If we explain these two outcomes according to the avoiding and returning cases in the labeling process, they should behave comparative quantity as they come from the same situation. The critical reason is the limited view of our sample video, the influencer’s position in the avoiding case may be off-screen, whereas the returning influencer always appears on screen. In other words, the distance between focus agent and influencer in the avoiding process is so long that we often cannot observe the influencer in the view. It is also an inevitable defect of this model caused by finite view of the fixed camera.

## 5. Influencer Recognition Model

After the transformation, we obtain a distribution over the influencers’ location in the frame of the focus agent, as represented by a 2-D kernel density estimator (KDE). The two dimensions are bearing and range. Furthermore, a video for visualization shows why pedestrians deflect in their trajectories apparently. We cannot regard pedestrians as moving obstacles on the account of intention, and they react intelligently [4]. Their trajectories have a few deflection points to divide trajectories. The deflections usually arise from the existence of spatial influencers (such as moving pedestrians). In its simplest form, the question we seek to answer is which pedestrian is the influencer of the focus agent in an obstacle-free environment. A kernel density estimator (KDE) is a non-parametric method to estimate the probability density function of a random variable. KDEs are applied widely when no parametric model distribution is known to be a good fit. A one-dimensional KDE is defined as

(1) $$f_{h}\left(x\right)=\frac{1}{nh}\sum_{i=1}^{n}K\left(\frac{x-x_{i}}{h}\right)$$

However, pedestrian data are positions, which are two-dimensional variables. For two-dimensional KDE, the hardest problem is how to select the metric $x-x_{i}$. We can divide this problem into two parts: selecting a coordinate system and designing an equivalent two-dimensional metric. Cartesian coordinates are conventional and intuitive. However, the Euclidean distance metric in Cartesian space incorrectly places influencers on all sides of the origin close together. Consider that such a metric would regard influencers near the origin in the first and third quadrants to be proximal, whereas our experience and results indicate they should not be confused. Typically, pedestrians with rightward deflections will not be influenced by a pedestrian in the left rear. (No such examples were found in the ETH data-set). We instead adopt a polar coordinate system, taking a modified Euclidean metric,

(2) $$m\left(\rho_{h},\theta_{h}\right)=\sqrt{\alpha {\left(\rho_{h}-\rho_{i}\right)}^{2}+\beta {\left(\theta_{h}-\theta_{i}\right)}^{2}},$$

where $\rho_{i}$ and $\theta_{i}$ are polar radius and polar angle of grid points, and $\rho_{h}$ and $\theta_{h}$ are polar radius and polar angle of sample points. α and β are the weight of polar radius and polar angle. By controlling α and β, we can adjust the proportion of the two variables.

Meanwhile, the selection of the kernel function *K* and smoothing parameter *h* also influence the KDE. Based on our experience, we chose a Gaussian function as kernel function, and we set the smoothing parameter h to 0.05. The two-dimensional KDE formula is

(3) $$f\left(\rho_{i},\theta_{i}\right)=\frac{1}{0.05n}\sum \frac{1}{\sqrt{2\pi }}\exp\left(-\frac{\alpha {\left(\rho_{h}-\rho_{i}\right)}^{2}+\beta {\left(\theta_{h}-\theta_{i}\right)}^{2}}{0.1}\right)$$

In addition, the edge effect in a KDE can impair performance. The estimate distribution near the frontier of the variable’s range will be incomplete if there are many data close to the boundary. The periodicity of angle eliminates the boundary in one dimension. To eliminate the influence of edge effect, we expand the data range to $\left[-360^{\circ },+360^{\circ }\right]$ and keep the estimating range as $\left[-180^{\circ },+180^{\circ }\right]$. Note that there is seldom an influencer located directly behind a pedestrian, so the discontinuity at $180^{\circ }$ is not a problem. Similarly, the edge effect for the polar radius is insignificant because there are few samples near 0 m or 10 m. The contour result is shown in the right of Figure 6.

## 6. Results

To calculate the prediction accuracy of the model, we contrast another data-set of ETH with this model. First, we select eligible pedestrians as focus agents with the following condition:

- The displacement should be bigger than 4 m;
- Their turning angle should be bigger than $5^{\circ }$;
- They should not gather to a group;
- Their fitting line should be correct.

**Figure 7:**
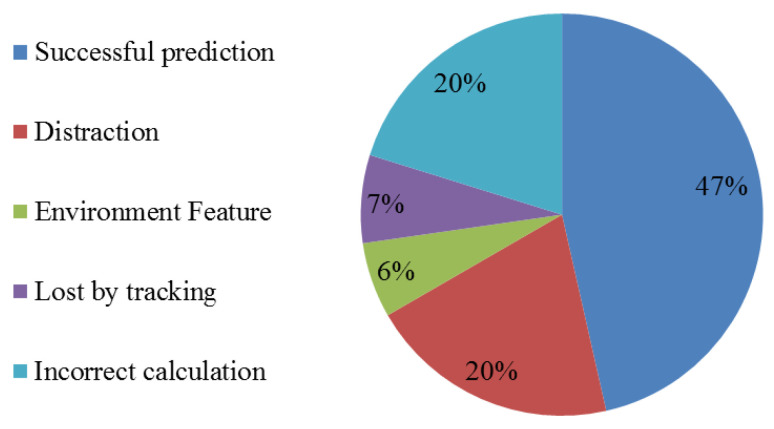
Prediction results as percentages. The most frequent outcome was a successful prediction. Distraction indicates failures because the focus pedestrian was not paying attention to surroundings or was otherwise not acting rationally. In roughly 20% of test cases (incorrect calculation), the model failed to predict the correct cause. Environment features are static obstacles not captured by the influencer recognition model (IRM).

For every eligible focus agent, we put the bearing and distance of other pedestrians into Equation (3). The pedestrian with the biggest probability should be the influencer to focus agent. There are 99 eligible samples total (Figure 7). Of these, 46 samples are predicted successfully; 20 samples are unpredictable as distraction; 6 samples fail because they are influenced by static obstacles; 7 samples’ real influencers are not in the view or tracked by the data-set; 20 samples are predicted incorrectly due to inaccuracies in our trained model.

The result is not ideal for several reasons. First, most errors caused by calculation derive from treating equally between avoiding and returning cases. But avoiding case happens when interaction begins while returning case happens when interaction ends. The result of the two cases may be influenced by condition changing (finite view of camera), so this outcome will make the model inaccurate and non-adaptive in different environments. Second, people always predict the future position of front pedestrians in avoiding cases. Actually avoiding pedestrian is affected by future trajectory of front pedestrians, IRM does not consider time sequence and it will lose influencers in the front sides of focus agent. Last, the training and testing data-sets of IRM must be carried in a low-obstacle environment. We have not found sufficient data-set for our method, and it brings inadequate robustness for IRM.

**Figure 8:**
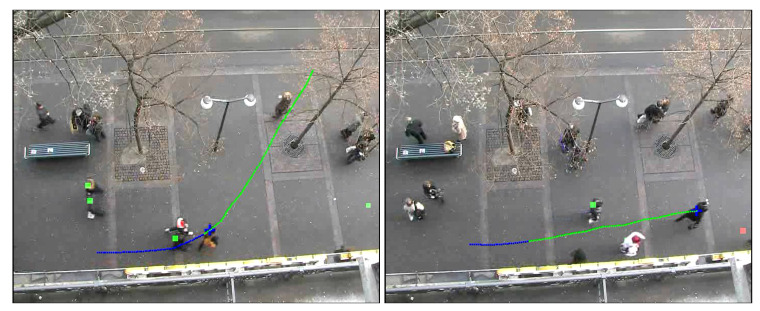
The visualization of the influencer model. The blue and green lines indicate two segments of the trajectory, each with consistent intention. The red square indicates the most likely influencer calculated by the influencer model (**left**). Sometimes, the model predicts an out-of-view pedestrian as designated by a red square at the edge of the screen (**right**).

We also visualize this model on testing video with symbols. The focus agent is shown by the blue cross and different parts of the trajectory are shown by different colors. The squares mean other pedestrians and their lengths correspond to their probabilities to be influencers. Finally, the biggest probability pedestrian is labeled by a red square, and others are labeled by green squares (left of Figure 8).

## 7. Discussion of IRM

### 7.1. Destination Prediction

According to the method proposed above, we illustrated the reason leading to pedestrian’s bend in a low-clutter environment. Furthermore, there is a deeper inspiration to explain what drives pedestrian to avoid a special pedestrian or obstacle. Presume that a focus agent’s trajectory is influencing by another pedestrian, this pedestrian must block the way of the focus agent. As the pedestrians are always driven by their intention, we can infer that the focus agent intends to reach the area blocked by the pedestrian.

**Figure 9:**
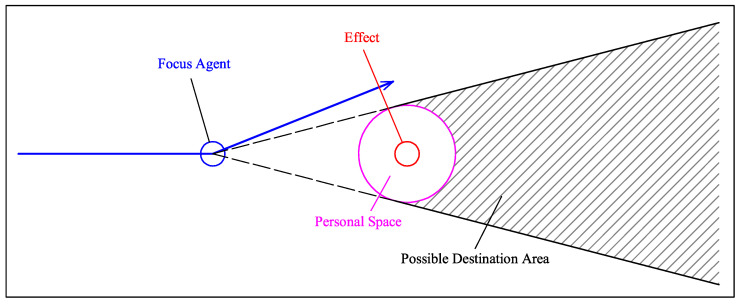
When the focus agent decides to turn, it is the approach to predict possible destination area by influencer recognition and its position. Blue line means fitting-line of trajectory, and purple circle illustrates the supposed avoidance area in focus agent’s mind.

Combining this idea and our influencer recognition model, it is possible that we can predict the destination by influencer’s position. As the avoiding case shown by Figure 9, the most important part is to ensure when the focus agent decides to turn. When the bend occurs, the influencer can be recognized by IRM. According to influencer’s position, setting a personal space of the influencer can make clear which area is avoided by the focus agent. Finally, the possible destination area can be inferred by the subgoal of the focus agent and the influencer’s position. In addition, introducing other variables (e.g., bearing) will be beneficial to make the result more accurate.

At present, it is an incomplete approach as uncertainty brought by real-time requirement, but it will be an outstanding prediction model in open space if we could improve the precondition and combine other information. In terms of time duration, destination prediction is a long-term and global method compared with short-term trajectory simulation.

### 7.2. To Discover GSEs or SSE

In a surrounding with people and static obstacles, two kinds of effect interact with pedestrians: general spatial effects (GSEs) and specific spatial effects (SSEs) [14]. GSEs are detectable and concrete obstacles (e.g., trees) while SSEs is abstract implication in the mind (e.g., sidewalk). The analysis of abnormal trajectories with no apparent reason to bend will inspire us to obtain latent information in the current environment.

After observing a focus agent with changing trajectory, assume an invisible GSE in the front of the focus agent and out of the view. If the invisible GSE has the biggest probability under the influencer recognition model, then we can infer that there is an invisible pedestrian influencing the focus agent’s trajectory out of view (right of Figure 8).

Furthermore, some SSEs’ characters can be inferred by long-term learning about abnormal trajectories. For example, pedestrians always bend to avoid the dangerous areas when they are passing cross, where pedestrians pay more attention to cars rather than persons. Collecting such trajectories without reasonable explanation may help to understand where is rejected by pedestrian’s subconscious. But it is difficult to conduct long-term learning only with IRM. For SSEs’ research, another method needs to be incorporated to understand environmental characteristics better. Learning motion patterns of people or building a cost-map of space [13] maybe good directions.

### 7.3. Limitations and Improvements

The method presented here provides an important first step towards addressing the problem of understanding pedestrian intent during obstacle avoidance. However, the method presents several limitations.

Most significantly, the model is currently trained only on pedestrian influencers, not static obstacles. The model may still predict static obstacles as influencers if aware of them, but the point approximation no longer applies to larger obstacles. People tend to walk along large obstacles (e.g., a curb or wall) with smooth curves (right in Figure 5). An environment with such features will greatly influence the trajectory in a manner that IRM cannot capture. A similar problem arises when a pedestrian is walking while distracted.

IRM only picks one influencer at a time to attribute the focus agent’s behavior. People standing in groups often collectively influence the focus agent, as combinations of unrelated people inadvertently standing in configurations will create complex influences on the focus agent. Such configurations would require IRM to account for multiple subgoals and recognize multiple influencers simultaneously to properly model the situation.

There are many directions for improvement. The primary objective of this research going forward is to collect a large pedestrian data-set and annotate it with trajectories calibrated in ground coordinates. Another objective is model improvement. The focus agent’s returning and avoiding processes must be analyzed separately. To enhance the predictive power of the model, we would like to introduce additional variables such as the turning angle of the focus agent.

## 8. The Navigation Probability Map

### 8.1. Our Data-Set in an Open Environment

**Figure 10:**
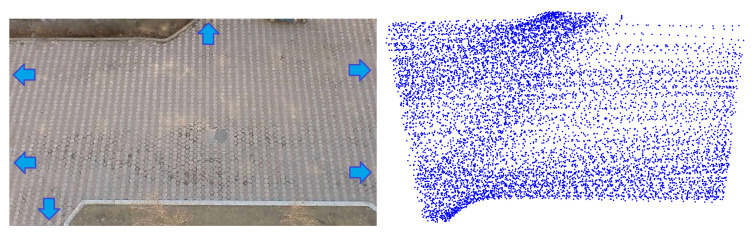
(**Left**): The environment where we collect data, the blue arrows show the most used entrances and exits. (**Right**): All the collected pedestrian data points.

In order to verify the further application of the IRM model, we collected a pedestrian data-set in an open environment. This is the entrance of a student dormitory with multiple destinations, and there is no obstruction or obstacle in the whole environment (left of Figure 10). We collected data when there were a lot of pedestrians.

**Figure 11:**
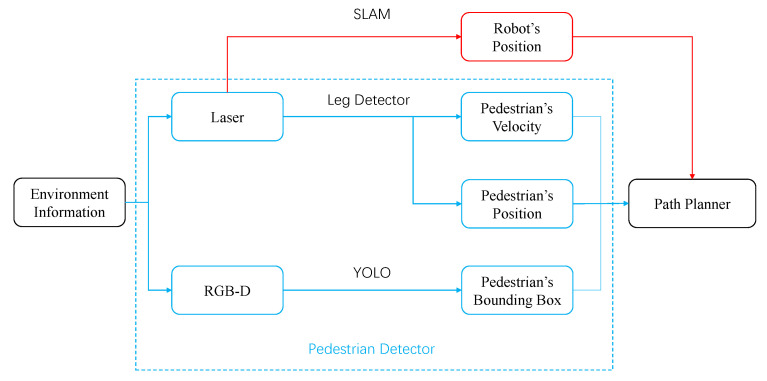
A robot sensor system that can provide the necessary input information for the path planner.

Our initial data is RGB image information, which is obtained by a bird’s-eye camera with a resolution of 1280 × 720 and a frame rate of 25 fps. This form of data is used to obtain environmental characteristics by analyzing pedestrian trajectories, and they are also easy to obtain in various situations with surveillance cameras. The robot can be equipped with laser and RGB-D cameras, and combine the information to complete pedestrian detection (Figure 11). The position detection of pedestrians can be implemented using the ROS (Robot Operating System) package “leg detector” [20], which will subscribe to the topic of laser and obtain the pedestrians’ positions and velocities in the environment. For obtaining the range of space occupied by pedestrians, it is difficult to infer the width of the pedestrian by only relying on the leg information. In order to increase the robot’s ability to perceive pedestrians, the depth camera and laser can be combined. YOLO can be used to extract the pedestrian bounding box to estimate the radius of the pedestrian.

For the bird’s-eye data, we used HOG, SVM and KCF to complete the collection of pedestrian data. The most widely used pedestrian recognition algorithm is the histogram of directional gradients proposed by Navneet Dalal in 2005. HOG is a feature descriptor used for object detection in image processing. It constructs features by calculating the histogram of the gradient direction of the local area of the unified image. HOG and SVM classifiers have been widely used in image recognition.

Although the HOG feature extraction and SVM classifier can complete pedestrian recognition, there are problems with using them for tracking. On the one hand, if the HOG feature extraction is performed on continuous 64 × 64 windows, the calculation for each frame will be large. On the other hand, The pedestrian window position obtained between different frames will vary greatly, which will result in great noise in the extracted trajectory data.

Because this is an unobstructed environment, all pedestrians must appear at the edge of the video first. First, we used HOG to continuously identify the position of the pedestrian at the edge of the screen. Once the positions of pedestrians are identified, their trajectories will be captured by the KCF algorithm. At last, we got a data-set with 361 pedestrians, the result is shown in the right of Figure 10. We publish our data-set on Github: https://github.com/spooncuber/Pedetriandata-set.

### 8.2. Probability Map

In a specific environment, the trajectory of pedestrians will follow certain rules, which are related to the characteristics of the environment. Pedestrians’ preferences can be preliminary inferred by analyzing the trajectories of pedestrians in an environment. But if there are many pedestrian trajectories, the entire environment may be densely covered, making the result worthless. However, it is possible to research the locations where pedestrians have more interactions with IRM.

**Figure 12:**
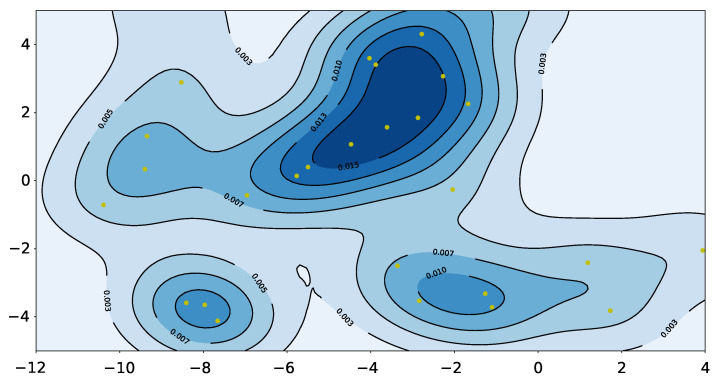
The probability map of our data-set. The yellow dots are the positions of pedestrians when they affect others’ trajectory. The darker the color of a certain location, the easier it is to affect other pedestrians.

We selected 30 target pedestrians in data-set according to the rules in Section 6, and used the IRM model to identify the location of the influencer when the focus pedestrian turned(the yellow dots in Figure 12). These positions are clustered at the top left and bottom of the map, because there are many pedestrians choosing the two corridors. More interactions will occur near these positions. In other words, when the robot moves to these darker areas, it is easier to affect pedestrians. So the robot needs to avoid these areas during path planning. According to this idea, we build a probability map that can describe the probability of pedestrians’ interaction (Figure 12).

### 8.3. Artificial Potential Field with Probability Map

The artificial potential field method is a commonly used method in path planning algorithms. It has the advantages of simple mathematical principle, small calculation amount, fast response speed, and good real-time control performance. The essence of the artificial potential field method is to establish a potential energy map, in which certain positions are adverse for the robot to approach. This concept fits well with the pedestrians’ influencer map mentioned in the previous section.

First, we establish the distribution of pedestrian influence through kernel density estimation. The darker color is the position where pedestrian interaction occurs intensively, as shown in Figure 12. Because there are no obstacles in this environment, it is not necessary to set repulsion for obstacles. For the attractive potential field generated by the target point, we use the traditional method to calculate it, as shown in the formula.

(4) $$U_{att}\left(q\right)=\frac{1}{2}\zeta d^{2}\left(q,q_{goal}\right)$$

ζ—Attractive gain

$d^{2}\left(q,q_{goal}\right)$—The distance between the current point *q* and goal.

**Figure 13:**
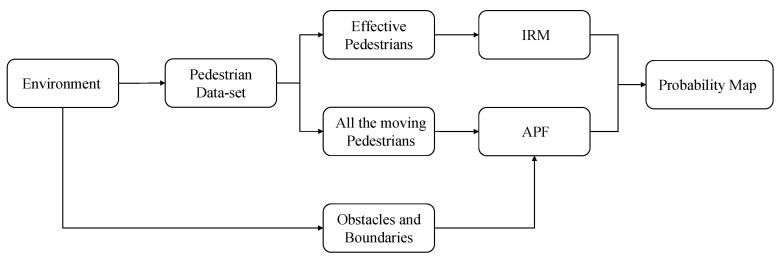
The process of getting the navigation probability map. If the environment has obstacles and boundaries, they can also be calculated by artificial potential field (APF).

**Figure 14:**
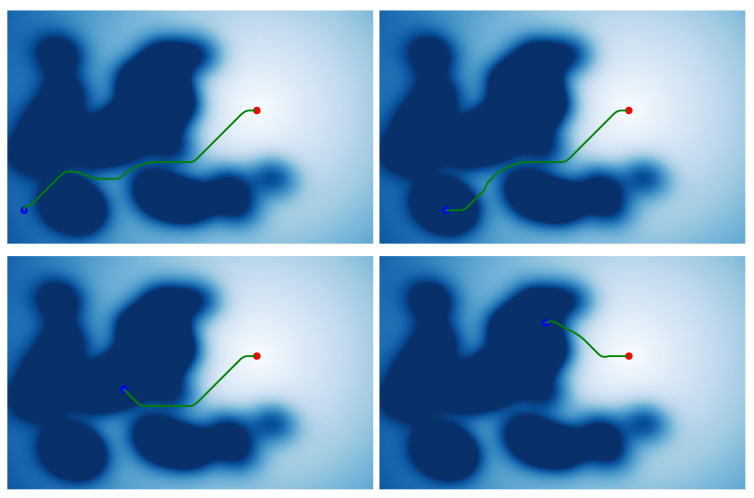
The planning results without pedestrians. This figure shows four results with different start points. (The blue dot is start point, and the red dot is end point.)

As shown in Figure 13, we can obtain the potential field map that combines the attraction field and the repulsive force field transformed by the probability map. The artificial potential field navigation using this map allows the robot to quickly leave areas that may block other pedestrians. The potential field map and navigation results are shown in Figure 14.

The right two results show that the robot will leave the dark area as soon as possible, where pedestrians are more likely to interact with each other, which means that pedestrians are more likely to be obstructed. The left two results show that when traversing these areas, the robot will try to avoid these areas or choose the line with the lowest impact to avoid affecting the movement of pedestrians.

### 8.4. Navigation Results in Data-Set

Finally, we will conduct a comparison simulation test between our method and the traditional method in our data-set with pedestrians. Because there are pedestrians in the data-set, pedestrians are treated as general obstacles in the artificial potential field method. The influence of pedestrians on the entire potential field is calculated as follows:

(5) $$U_{rep}\left(q\right)=\left\{\begin{array}{cc} \frac{1}{2}\eta \left(\frac{1}{D(q)}-\frac{1}{Q^{*}}\right), & D\left(q\right)\leq Q^{*}\\ 0, & D\left(q\right)>Q^{*} \end{array}\right.$$

D(q)—Distance between point *q* and its nearest pedestrian

η—Repulsion gain

$Q^{*}$—Pedestrian repulsion distance threshold

**Figure 15:**
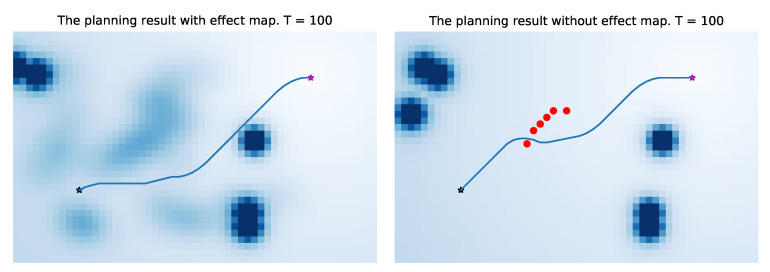
Comparison result of integration method and traditional artificial potential field method. The the black star is start point, and the purple start is end point. The dark circles are pedestrians and the red dots are positions of pedestrians at the time of collision with robot.

**Figure 16:**
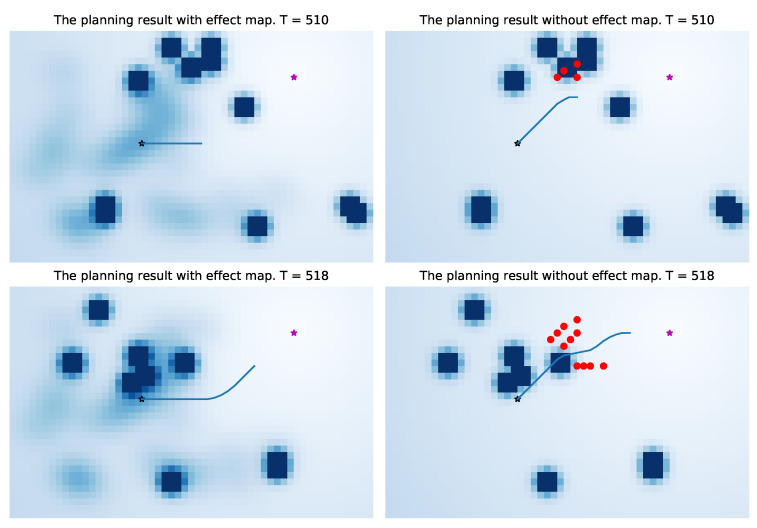

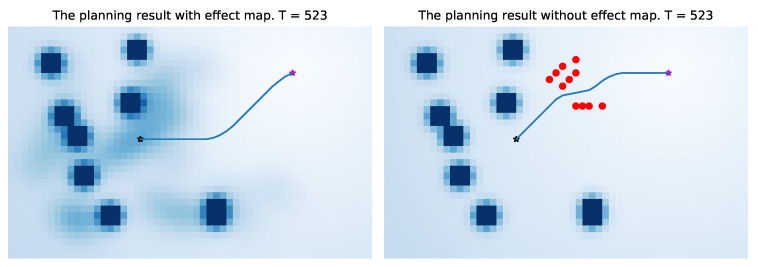
Comparison of a complete dynamic process. The three images on the right show that if the robot does not understand the environmental characteristics, its navigation behavior is more likely to conflict with pedestrians.

In Figure 15, the left one is the navigation result of the artificial potential field method incorporating the probability map, and the right one is the navigation result of the traditional artificial potential field method. It can be seen from the results that in our method, the robot will leave the position that may affect the pedestrian as soon as possible, so there is less conflict with the pedestrian. Under the same condition, the traditional artificial potential field method is more likely to affect pedestrians without additional information due to the characteristics of the greedy algorithm(the red dots are positions of pedestrians at the time of collision with the robot). A complete dynamic process is shown in Figure 16. Because the entire environment is equivalent to the traditional APF algorithm, it will not optimize the robot’s path selection based on environmental characteristics, which makes the robot easier to conflict with pedestrians (the right images in Figure 16).

In order to validate our method in the entire data-set, we compare the two methods at different times. For the end point and start point shown in Figure 15, we run the two methods every 40 seconds, and record the collision with pedestrians. The main evaluation index is the number and times of collisions with pedestrians. “Number of Pedestrian Collisions” reflects the degree of collisions between the robot and pedestrians, and “Times of Pedestrian Collisions” (total times of pedestrian collisions at each moment) reflects the robot’s ability to handle conflicts. We verify a total of 14 times (0 s, 40 s, …, 520 s in our data-set), the results are shown in Table 1. Our method is better than the artificial potential field method without probability map by 19.2% in the “the number of pedestrian collisions”, and is 16.9% better than the APF method in the “the times of pedestrian collision”.

The method we propose has good real-time performance on navigation problems. And our method has good portability, with platform and hardware independence. The idea of navigation probability map can also be well transferred to other navigation methods. At the same time, this idea will not rely on specific sensors and system architecture. As long as a sensor system can accurately detect the location of pedestrians, it can learn the navigation probability map through the pedestrian trajectory. Although our method outperforms traditional methods in most cases, this method uses few types of information. The robot only uses the current information of the pedestrian, which can be improved in future research.

### 8.5. Possible Applications

Due to the simplicity and generalization of the concept of probability map, it can be combined with some other robot navigation methods. These methods require a basic environment and pedestrian perception, and a major planning system (such as APF). The collision-free navigation algorithm [9] is based on an integrated representation of the information about the environment. If it is applied to a dense pedestrian scene, the probability map can be combined with its navigation method in a certain way. Another navigation method [22] can avoid a pedestrian approaching from the front and to pass him/her by, while preserving the public distance of personal space. For this method, the integration of other environment information is also necessary. Even for navigation algorithms that explore special environments (such as narrow corridor), pedestrians’ preferences are very meaningful (such as right-handed rules).

## 9. Conclusions

In this paper, we established the influencer recognition model (IRM), a method to reveal the change of pedestrian’s trajectory based on real pedestrian data-set. The model can identify influencers correctly in half. Most errors are caused by pedestrians’ confusing intent or tracking errors of data-set. The process of IRM is fast and easy to reproduce.

Then we applied IRM to a specific environment scene and obtained the environment’s probability map, which describes where pedestrians are more likely to interact. For robots, moving in these areas is not suitable for social navigation, because it will easily affect pedestrians. Based on this principle, we combined the traditional artificial potential field method to propose a new navigation method. This method is simple and fast to execute and make better social navigation results after learning the environmental characteristics. Our method is better than the traditional method by 19.2% in the “the number of pedestrian collisions”. The idea of navigation probability map can be applied in an environment with few obstacles but high dynamics. The information it reveals reflects a navigation rule that the robot should follow.

## Figures and Tables

**Table 1:** The method comparison results. (APF means Artificial Potential Field).

Method	Number of Pedestrian Collisions	Times of Pedestrian Collisions
APF	52	148
our method	42	123

## Data Availability

Publicly available datasets were analyzed in this study. This data can be found here: https://github.com/spooncuber/PedetrianDataset.
